# Specialty‐Based Disparities in Biologic Use and Retention in Psoriatic Arthritis: A Nationwide Korean Claims Analysis

**DOI:** 10.1111/1756-185x.70756

**Published:** 2026-06-26

**Authors:** Bon San Koo, Ye‐Jee Kim, Yeo‐Jin Lee, Yong‐Gil Kim, Tae‐Hwan Kim

**Affiliations:** ^1^ Division of Rheumatology, Department of Internal Medicine Inje University Ilsan Paik Hospital, Inje University College of Medicine Goyang‐si Gyeonggi‐do South Korea; ^2^ Department of Clinical Epidemiology and Biostatistics, Asan Medical Center University of Ulsan College of Medicine Seoul South Korea; ^3^ Department of Rheumatology, Asan Medical Center University of Ulsan College of Medicine Seoul South Korea; ^4^ Department of Rheumatology Hanyang University Hospital for Rheumatic Diseases Seoul South Korea

**Keywords:** biologic agents, national health insurance data, prescription patterns, psoriatic arthritis

## Abstract

**Aim:**

Biologic agents play a pivotal role in controlling both articular and cutaneous manifestations of psoriatic arthritis (PsA). Although international guidelines emphasize the appropriate use of biologics, Korea lacks standardized treatment protocols tailored for PsA across specialties. We therefore aimed to examine specialty‐specific prescription patterns and treatment retention using nationwide claims data.

**Methods:**

Health Insurance Review and Assessment (HIRA) claims data from 2011 to 2022 were analyzed, including patients with PsA who initiated treatment with their first biologic agent. Patients with concurrent autoimmune diseases were excluded. Trends in diagnosis, prescription patterns, and retention were analyzed according to medical specialty.

**Results:**

Of the 2406 patients with PsA, 63.0% were diagnosed in dermatology departments and 32.8% in internal medicine departments. Most biologics were prescribed in the dermatology department (80.2%), while 17.8% were prescribed in the internal medicine department. Since 2016, dermatology has exhibited a marked increase in both PsA diagnoses and biologic prescriptions. Interleukin‐23 (IL‐23) inhibitors were the most frequently prescribed class (62.0%), followed by IL‐17 inhibitors (21.9%) and tumor necrosis factor inhibitors (TNFi) (16.1%). Drug retention analysis revealed that dermatology was associated with a significantly higher risk of biologic discontinuation than internal medicine (hazard ratio = 1.40, 95% confidence interval 1.11–1.78).

**Conclusion:**

The predominance of PsA diagnosis and biologic initiation in dermatology indicates substantial specialty‐specific differences in practice. This pattern warrants careful evaluation and supports the development of standardized, evidence‐based guidelines across specialties to promote appropriate care.

## 1 Introduction

Psoriatic arthritis (PsA) is a chronic inflammatory arthritis associated with psoriasis. It shares genetic and clinical characteristics with other forms of spondyloarthritis, situating it within the broader spondyloarthritis spectrum [1, 2]. Globally, the prevalence of PsA is approximately 112 per 100 000 adults, with higher prevalence rates in Europe and North America than in Asia or South America [3]. Although the prevalence of PsA in Korea has not been well established, the incidence among patients with psoriasis has steadily increased, reaching 5.01 cases per 1000 person‐years in 2020 [4].

Approximately 30% of patients with psoriasis develop PsA, and up to 15% of those managed in dermatologic settings may have undiagnosed PsA [5]. This diagnostic gap underscores the risk of under‐recognition, particularly when skin symptoms are mild or when clinical attention remains focused on cutaneous manifestations [6]. Delayed diagnosis and undertreatment of joint involvement further exacerbate disease burden [7, 8, 9]. These challenges emphasize the need to enhance clinician awareness and promote interdisciplinary collaboration among dermatologists, rheumatologists, and other specialists in the diagnosis and management of PsA.

Biologic agents have demonstrated substantial therapeutic efficacy in both psoriasis and PsA. Since the introduction of tumor necrosis factor inhibitors (TNFi) in the early 2000s, followed by the approval of interleukin‐17 (IL‐17) and interleukin‐23 (IL‐23) inhibitors for PsA in Korea, treatment outcomes for these conditions have markedly improved [10]. However, concerns persist regarding the potential overutilization of biologics in PsA because of ambiguity in diagnostic criteria and biologic indications [6, 10].

Such ambiguity may result in inappropriate use of costly medications and hinder consistent monitoring of treatment response and long‐term disease control. Variations in diagnostic approaches and therapeutic strategies across medical specialties, such as dermatology and rheumatology, can lead to patient confusion and complicate clinical decision‐making. Ultimately, these disparities may strain national healthcare resources and adversely affect patients' quality of life.

A health services perspective on biologic prescription patterns in PsA is therefore warranted. We aimed to examine the real‐world use of biologics in Korean patients with PsA by analyzing nationwide data from the Health Insurance Review and Assessment Service (HIRA), with an emphasis on variations by hospital type and medical specialty. Additionally, we analyzed biologic drug retention rates to gain insights into their long‐term use in clinical practice.

## 2 Materials and Methods

### 2.1 Patients

This study utilized data obtained from the HIRA, which included patients aged 20–85 years who were newly diagnosed with PsA between January 1, 2011, and June 30, 2022. Patients who had been diagnosed with PsA within 1 year before their index date were excluded. Eligible cases were identified using the corresponding ICD‐10 codes for PsA (M07.0, M07.2, and M07.3) and the rare disease registration code (V237). Patients with diagnostic codes for systemic lupus erythematosus, rheumatoid arthritis, Behçet's disease, ankylosing spondylitis, or inflammatory bowel disease were excluded. Additionally, patients who did not revisit the hospital within 3 months of their initial diagnosis were excluded (Table S1).

Analysis of biologic agents was restricted to the first biologic prescription for each patient. To assess biologic drug retention, continuation was defined as a prescription of the same biologic agent within 180 days of the last prescription, whereas discontinuation was defined as switching to a different biologic agent, absence of any biologic prescription for more than 180 days, or prescription of the same biologic agent beyond 180 days after the last prescription.

### 2.2 Clinical Characteristics of Patients

Patient characteristics included age (categorized into 10‐year intervals starting from 20 years), sex, year of diagnosis, and prescription of biologic agents. Prescription data for biologic agents approved and covered by health insurance in South Korea were collected, including TNFi (e.g., adalimumab, etanercept, golimumab, and infliximab), IL‐17i (e.g., secukinumab and ixekizumab), and IL‐23i (e.g., ustekinumab, guselkumab, and risankizumab; Table S2). The hospital types and medical specialties responsible for diagnoses and prescriptions were also investigated. Comorbidities, including dyslipidemia, hypertension, ischemic heart disease, stroke, asthma, and chronic obstructive pulmonary disease (COPD), were identified using diagnostic codes from 1 year before the index date, and the Charlson Comorbidity Index (CCI) was calculated based on these data.

### 2.3 Statistical Analysis

Continuous variables were expressed as means (standard deviations), whereas categorical variables were expressed as numbers (percentages). Statistical significance was set at *p* < 0.05, with all hazard ratios (HRs) reported alongside 95% confidence intervals (CIs). For basic statistical analyses, *t*‐tests and chi‐square tests were employed. Retention rates were analyzed using Kaplan–Meier survival curves, which were plotted for each biologic agent and stratified by specialty. The median survival time (in days), defined as the point at which 50% of the patients discontinued the biologic agent, was also calculated. To identify factors associated with the retention rate of biologic agents, HRs and adjusted HRs (aHRs) were calculated. The multivariate model for the aHRs included variables such as age, sex, and comorbidities.

Statistical analyses were conducted using SAS Enterprise Guide (version 6.1; SAS Institute Inc., Cary, NC, USA) and R (version 3.5.2; R Foundation for Statistical Computing, Vienna, Austria). Additional graphs were generated using Microsoft Excel.

## 3 Results

**FIGURE 1:**
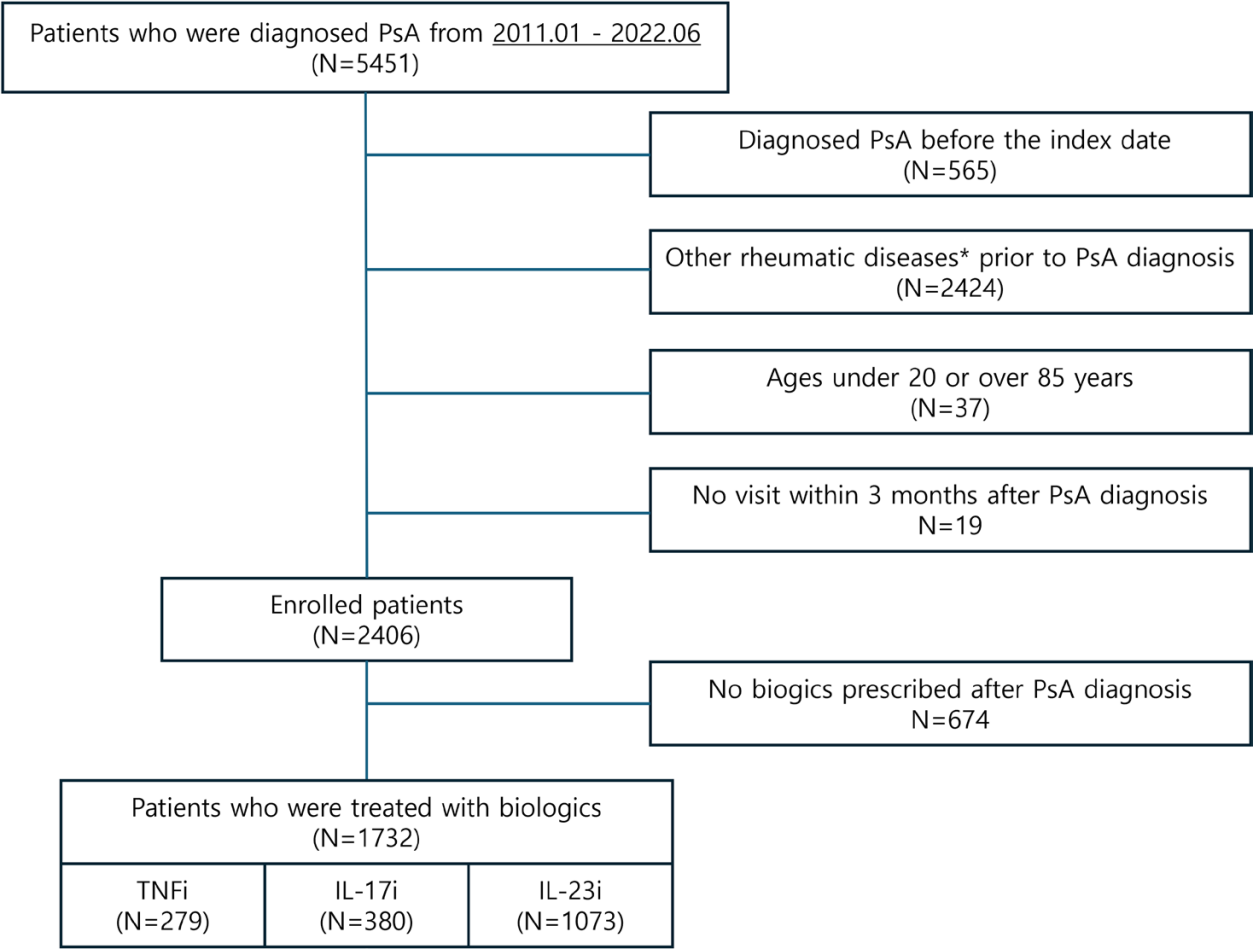
Flowchart illustrating the process of patient selection. *Other rheumatic diseases as exclusion criteria include systemic lupus erythematosus, rheumatoid arthritis, Bechet’s disease, ankylosing spondylitis, and inflammatory bowel disease.

From a total of 5451 patients with PsA‐related diagnostic codes, 2406 newly diagnosed patients were enrolled. Among them, 674 did not receive a biologic agent prescription, whereas 1732 patients did. The distribution of biologic agent prescriptions was as follows: TNFi (*n* = 279), IL‐17i (*n* = 380), and IL‐23i (*n* = 1073) (Figure 1). As summarized in Table 1, the most common age group for both diagnosis and biologic agent prescription was 40–59 years (49.2% and 47.7%, respectively), and male patients comprised the majority of the cohort. Over 95% of the patients received both their PsA diagnosis and biologic agent prescription at a tertiary or general hospital. Notably, the majority of PsA diagnoses were made in the dermatology department (63.0%), followed by the internal medicine department (32.8%). For biologic prescriptions, the dermatology and internal medicine departments accounted for 80.2% and 17.8% of the prescriptions, respectively.

**TABLE 1:** Baseline characteristics of study participants diagnosed with PsA.

Variable	PsA diagnosis (*n* = 2406)	(%)	Prescribed biologics (*n* = 1732)	(%)
Age group (years)				
20–29	256	10.6	210	12.1
30–39	536	22.3	408	23.6
40–49	584	24.3	417	24.1
50–59	599	24.9	408	23.6
60–69	314	13.1	217	12.5
70–84	117	4.9	72	4.2
Sex				
Men	1533	63.7	1130	65.2
Women	873	36.3	602	34.8
Year of PsA diagnosis				
2012–2015	353	14.7	194	11.2
2016–2018	898	37.3	639	36.9
2019–2022.06	1155	48.0	899	51.9
Type of medical institution				
Tertiary hospital	1270	52.8	859	49.6
General hospital	1032	42.9	827	47.7
Hospital	19	0.8	8	0.5
Clinic	85	3.5	38	2.2
Clinical specialty				
Internal medicine	788	32.8	309	17.8
Dermatology	1515	63.0	1389	80.2
Orthopedics	74	3.1	32	1.8
Others	29	1.2	2	0.1
Comorbidities (1 year prior)				
Dyslipidemia	1128	46.9	817	47.2
Hypertension	656	27.3	455	26.3
Diabetes mellitus	399	16.6	271	15.6
Ischemic heart disease	147	6.1	96	5.5
Stroke	55	2.3	33	1.9
Asthma	206	8.6	146	8.4
COPD	16	0.7	9	0.5
Charlson comorbidity index				
0	1054	43.8	778	44.9
1	665	27.6	489	28.2
2+	687	28.6	465	26.8
Biologic agents used				
Adalimumab	226	9.4	226	13.0
Etanercept	44	1.8	44	2.5
Infliximab	26	1.1	26	1.5
Golimumab	31	1.3	31	1.8
Secukinumab	430	17.9	430	24.8
Ixekizumab	189	7.9	189	10.9
Ustekinumab	609	25.3	609	35.2
Guselkumab	671	27.9	671	38.7
Risankizumab	204	8.5	204	11.8

Abbreviations: CCI, Charlson comorbidity index; COPD, chronic obstructive pulmonary disease; PsA, psoriatic arthritis.

### 3.1 Trends in Diagnosis and Prescription of PsA

**FIGURE 2:**
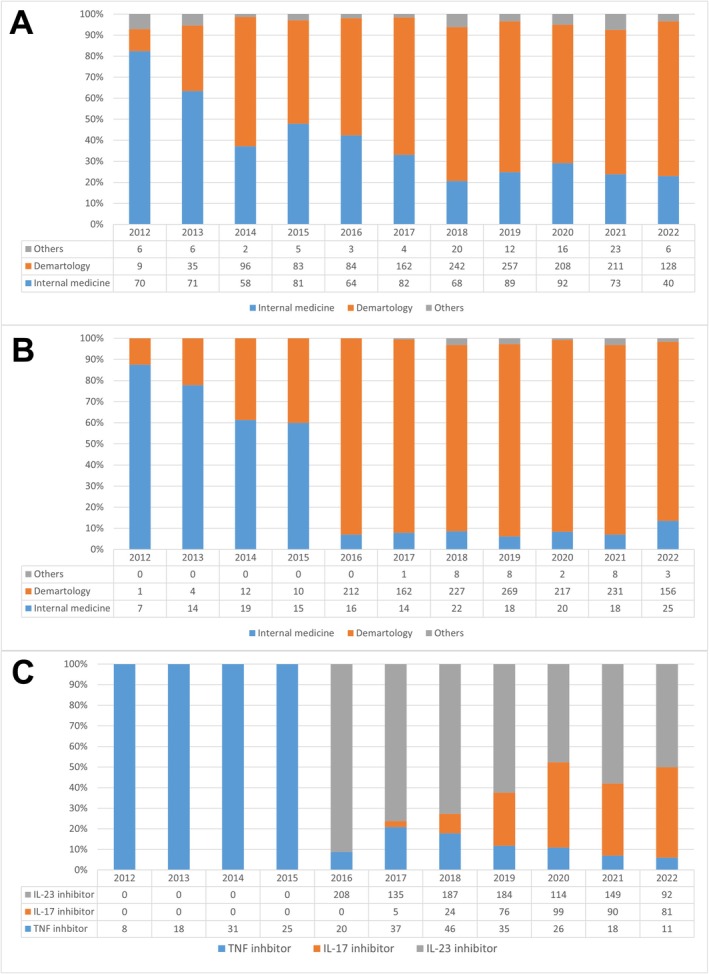
Trends in the diagnosis of psoriatic arthritis (PsA) and initiation of biologic agents. (A) Annual number of PsA diagnoses by department (*n* = 2406). (B) Annual number of patients with PsA initiating biologic agents by department (*n* = 1732). (C) Annual number of patients initiating each class of biologic agents (*n* = 1732).

Figure 2 illustrates longitudinal trends in PsA diagnoses and biologic prescriptions. As demonstrated in Figure 2A, the number of PsA diagnoses made by the internal medicine department remained relatively stable, whereas those made by the dermatology and other departments increased steadily after 2012.

As illustrated in Figure 2B, the number of prescriptions rose sharply in 2016 and thereafter stabilized, maintaining a trend comparable to the yearly incidence of newly diagnosed patients, indicating a trend toward early initiation of biologics. Figure 2C illustrates changes in the types of biologics prescribed. IL‐23 inhibitor prescriptions rose sharply in 2016, followed by a gradual increase in IL‐17 inhibitor prescriptions. These newer biologic classes were increasingly adopted over time, reflecting evolving real‐world prescribing preferences. Overall, these findings indicate a significant shift not only in PsA diagnosis, with growing dermatology predominance, but also in its treatment, particularly in the early adoption of new biologic agents in dermatology practice.

### 3.2 Characteristics of Biologic Agent Prescription

The median interval between PsA diagnosis and biologic agent prescription was 157 days (IQR: 17–448) (Table 2). Similarly, the median time for TNFi was 157 days (17–448), whereas it was substantially shorter for IL‐17i (median 0 days [0–84]) and IL‐23i (median 0 days [0–87]), indicating that these drugs were frequently prescribed immediately after diagnosis. Regarding hospital type, TNFi and IL‐17i were most frequently prescribed at tertiary hospitals (68.5% and 52.6%, respectively), whereas IL‐23i was most frequently prescribed at general hospitals (52.7%). Prescribing patterns also varied markedly by department: TNFi were primarily prescribed by the internal medicine department (60.2%), whereas IL‐17i and IL‐23i were almost exclusively prescribed by the dermatology department (94.5% and 96.8%, respectively). Differences in comorbidities were observed for dyslipidemia and asthma, and the CCI also differed across biologic treatment groups.

**TABLE 2:** Distribution of biologic agents.

Variable	Total (*n* = 1732)	TNFi (*n* = 279)	IL‐17i (*n* = 380)	IL‐23i (*n* = 1073)	*p*
Days to biologic initiation, median (IQR)	157 (17–448)	157 (17–448)	0 (0–84)	0 (0–87)	—
Mean ± SD	188.3 ± 427.2	381.8 ± 620.3	149.9 ± 392.1	151.6 ± 359	
Min–max	0–3550	0–3550	0–2635	0–3062	
Year of biologic initiation, *n* (%)					
2012–2015	82 (4.7)	82 (29.4)	0 (0.0)	0 (0.0)	
2016–2018	662 (38.2)	103 (36.9)	29 (7.6)	530 (49.4)	
2019–2022.06	988 (57.0)	94 (33.7)	351 (92.4)	543 (50.6)	
Age group (years), *n* (%)					0.058
20–29	202 (11.7)	23 (8.2)	50 (13.2)	129 (12.0)	
30–39	395 (22.8)	78 (28.0)	83 (21.8)	234 (21.8)	
40–49	418 (24.1)	78 (28.0)	84 (22.1)	256 (23.9)	
50–59	411 (23.7)	65 (23.3)	89 (23.4)	257 (24.0)	
60–69	306 (17.7)	35 (12.5)	74 (19.5)	197 (18.4)	
Sex, *n* (%)					0.074
Men	1130 (65.2)	173 (62.0)	235 (61.8)	722 (67.3)	
Women	602 (34.8)	106 (38.0)	145 (38.2)	351 (32.7)	
Year of PsA diagnosis, *n* (%)					< 0.0001
2012–2015	194 (11.2)	91 (32.6)	1 (0.3)	102 (9.5)	
2016–2018	639 (36.9)	125 (44.8)	55 (14.5)	459 (42.8)	
2019–2022.06	899 (51.9)	63 (22.6)	324 (85.3)	512 (47.7)	
Type of medical institution, *n* (%)					< 0.0001
Tertiary hospital	860 (49.7)	191 (68.5)	200 (52.6)	469 (43.7)	
General hospital	826 (47.7)	83 (29.7)	178 (46.8)	565 (52.7)	
Hospital	9 (0.5)	4 (1.4)	2 (0.5)	3 (0.3)	
Clinic	37 (2.1)	1 (0.4)	0 (0.0)	36 (3.4)	
Clinical specialty, *n* (%)					< 0.0001
Internal medicine	192 (11.1)	168 (60.2)	20 (5.3)	4 (0.4)	
Dermatology	1509 (87.1)	111 (39.8)	359 (94.5)	1039 (96.8)	
Others	31 (1.8)	0 (0.0)	1 (0.3)	30 (2.8)	
Comorbidities (1 year prior), *n* (%)					
Dyslipidemia	873 (50.4)	136 (48.7)	166 (43.7)	571 (53.2)	0.005
Hypertension	481 (27.8)	90 (32.3)	98 (25.8)	293 (27.3)	0.160
Diabetes mellitus	297 (17.1)	49 (17.6)	67 (17.6)	181 (16.9)	0.925
Ischemic heart disease	93 (5.4)	14 (5.0)	24 (6.3)	55 (5.1)	0.650
Stroke	37 (2.1)	6 (2.2)	5 (1.3)	26 (2.4)	0.439
Asthma	154 (8.9)	36 (12.9)	25 (6.6)	93 (8.7)	0.017
COPD	12 (0.7)	4 (1.4)	4 (1.1)	4 (0.4)	0.103
Charlson comorbidity index, *n* (%)					0.016
0	705 (40.7)	90 (32.3)	158 (41.6)	457 (42.6)	
1	502 (29.0)	86 (30.8)	117 (30.8)	299 (27.9)	
2+	525 (30.3)	103 (36.9)	105 (27.6)	317 (29.5)	

Abbreviations: COPD, chronic obstructive pulmonary disease; IL‐17i, interleukin‐17 inhibitors; IL‐23i, interleukin‐23 inhibitors; IQR, interquartile range; PsA, psoriatic arthritis; SD, standard deviation; TNFi, tumor necrosis factor inhibitors.

### 3.3 Retention Rates of Biologic Agents

**FIGURE 3:**
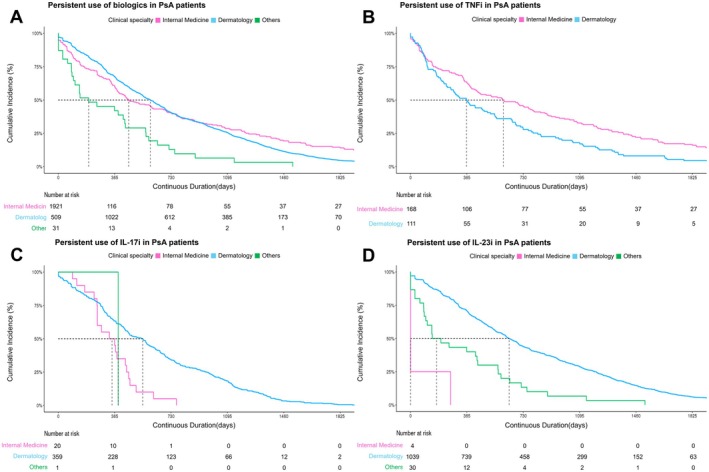
Comparison of biologic drug retention rates by medical specialty. Kaplan–Meier survival curves illustrating retention rates of biologic agents by specialty. (A) Overall retention rates across all biologic classes. (B) Retention rates for tumor necrosis factor inhibitors (TNFi). (C) Retention rates for interleukin‐17 inhibitors (IL‐17i). (D) Retention rates for interleukin‐23 inhibitors (IL‐23i).

The retention rate of each biologic agent is illustrated in Figure 3. When all biologic agents were considered together, the time to 50% discontinuation was the longest in the dermatology department, followed by the internal medicine and other departments. However, as illustrated in Figure 3A, the retention curves for dermatology and internal medicine intersected around day 800, precluding definitive conclusions about which department achieved superior long‐term persistence. The retention duration of TNFi was longer in the internal medicine department than in other departments, whereas for both IL‐17i and IL‐23i, it was longer in the dermatology department than in other departments (Figure 3B–D, respectively). The exact time to 50% discontinuation of each biologic agent by department is presented in Table S3. Notably, sample sizes were relatively balanced between internal medicine and dermatology for TNFi in Figure 3B, allowing meaningful comparison; conversely, sample sizes were substantially skewed toward dermatology for IL‐17i and IL‐23i in Figure 3C,D. This imbalance limits the interpretability of interdepartmental comparisons for these agents.

Figure S1A illustrates the comparison of biologic retention rates across departments, highlighting that IL‐23 inhibitors exhibited the longest median time to discontinuation. However, the retention curves of IL‐23i and TNFi intersect at approximately 1200 days, precluding a definitive conclusion regarding long‐term persistence superiority between the two classes. Moreover, substantial differences in sample sizes among biologic agents hinder the direct comparison of retention superiority. Figure S1B,C illustrate department‐specific retention patterns: in internal medicine, TNFi demonstrated the longest time to 50% discontinuation, whereas in dermatology, IL‐23i demonstrated the longest time. However, because of unequal sample sizes and overlapping curves, it remains difficult to determine which treatment has superior persistence in either specialty.

### 3.4 Hazard Ratios for Drug Retention

In the univariate analysis, IL‐17 inhibitors were associated with a significantly higher risk of discontinuation than that observed with IL‐23 inhibitors (HR = 1.39, 95% CI 1.24–1.57, *p* < 0.001), and this association remained consistent across all multivariable models (Table 3). Conversely, TNF inhibitors did not differ significantly in persistence compared with IL‐23 inhibitors in any model. Older age (60–84 years) was associated with an increased risk of discontinuation.

**TABLE 3:** Cox proportional hazards models for biologic discontinuation among PsA patients initiating a first biologic agent.

Variable	Univariate			Multivariable 1^a^			Multivariable 2^b^			Multivariable 3^c^			Multivariable 4^d^		
	HR	95% CI	*p*	HR	95% CI	*p*	HR	95% CI	*p*	HR	95% CI	*p*	HR	95% CI	*p*
Type of biologics															
TNFi	0.90	0.79–1.03	0.136	1.17	0.95–1.43	0.142	1.17	0.95–1.43	0.138	1.17	0.95–1.43	0.139	1.17	0.95–1.44	0.135
IL‐17i	1.39	1.24–1.57	< 0.001	1.47	1.31–1.66	< 0.001	1.47	1.30–1.66	< 0.001	1.45	1.29–1.64	< 0.001	1.45	1.29–1.64	< 0.001
IL‐23i (ref)	1.00	—	—	1.00	—	—	1.00	—	—	1.00	—	—	1.00	—	—
Sex															
Male (ref)	1.00	—	—	—	—	—	—	—	—	—	—	—	—	—	—
Female	1.05	0.95–1.16	0.299	—	—	—	—	—	—	—	—	—	—	—	—
Age group (years)															
20–59 (ref)	1.00	—	—	1.00	—	—	1.00	—	—	1.00	—	—	1.00	—	—
60–84	1.25	1.10–1.41	< 0.001	1.17	1.03–1.34	0.019	1.17	1.03–1.34	0.018	1.24	1.10–1.41	0.001	1.24	1.10–1.41	0.001
Medical institution															
Tertiary hospital (ref)	1.00	—	—	1.00	—	—	1.00	—	—	1.00	—	—	1.00	—	—
General hospital	1.13	1.03–1.25	0.011	1.12	1.01–1.23	0.027	1.12	1.01–1.23	0.026	1.13	1.02–1.24	0.017	1.13	1.02–1.24	0.016
Hospital	1.98	1.02–3.81	0.042	2.64	1.34–5.21	0.005	2.42	1.25–4.71	0.009	2.52	1.28–4.96	0.008	2.33	1.20–4.53	0.012
Clinic	2.64	1.89–3.67	< 0.001	3.98	2.04–7.77	< 0.001	2.89	2.07–4.04	< 0.001	3.90	2.00–7.61	< 0.001	2.89	2.07–4.04	< 0.001
Specialty															
Internal medicine (ref)	1.00	—	—	1.00	—	—	—	—	—	1.00	—	—	—	—	—
Dermatology	1.30	1.11–1.52	0.001	1.42	1.12–1.81	0.004	—	—	—	1.40	1.11–1.78	0.005	—	—	—
Others	2.92	1.99–4.29	< 0.001	0.97	0.46–2.05	0.929	—	—	—	0.98	0.46–2.08	0.952	—	—	—
Department															
Internal medicine (ref)	—	—	—	—	—	—	1.00	—	—	—	—	—	1.00	—	—
Non‐internal medicine	—	—	—	—	—	—	1.41	1.11–1.79	0.004	—	—	—	1.40	1.10–1.77	0.006
Comorbidities (1 year prior)															
Dyslipidemia	1.12	1.02–1.23	0.020	1.08	0.98–1.21	0.135	1.08	0.98–1.21	0.134	—	—	—	—	—	—
Hypertension	1.13	1.02–1.26	0.021	1.07	0.95–1.21	0.277	1.07	0.94–1.20	0.314	—	—	—	—	—	—
Diabetes mellitus	1.12	0.99–1.27	0.084	1.00	0.87–1.15	0.987	1.00	0.87–1.15	0.975	—	—	—	—	—	—
Ischemic heart disease	1.22	0.99–1.50	0.066	1.13	0.91–1.41	0.269	1.13	0.91–1.41	0.261	—	—	—	—	—	—
Stroke	1.02	0.74–1.41	0.912	—	—	—	—	—	—	—	—	—	—	—	—
Asthma	1.01	0.85–1.19	0.923	—	—	—	—	—	—	—	—	—	—	—	—
COPD	1.60	0.91–2.82	0.106	—	—	—	—	—	—	—	—	—	—	—	—
Charlson comorbidity index															
0 (ref)	1.00	—	—	—	—	—	—	—	—	—	—	—	—	—	—
1	1.01	0.90–1.13	0.892	—	—	—	—	—	—	—	—	—	—	—	—
2+	1.10	0.99–1.24	0.090	—	—	—	—	—	—	—	—	—	—	—	—

*Note:* Clinical specialty reflects the specialty code recorded in claims (internal medicine, dermatology, others). Department is a dichotomized variable (internal vs. non‐internal medicine) used to account for potential misclassification of rheumatology‐coded care within internal medicine in claims data.

Abbreviations: CI, confidence interval; COPD, chronic obstructive pulmonary disease; HR, hazard ratio; IL‐17i, interleukin‐17 inhibitors; IL‐23i, interleukin‐23 inhibitors; PsA, psoriatic arthritis; TNFi, tumor necrosis factor inhibitors.

^a^ Multivariable model 1 adjusted for age group, sex, type of medical institution, clinical specialty, and comorbidities (dyslipidemia, hypertension, diabetes mellitus, ischemic heart disease).

^b^ Multivariable model 2 adjusted for age group, sex, type of medical institution, department (internal vs. non‐internal), and the same comorbidities.

^c^ Multivariable model 3 adjusted for age group, type of medical institution, and clinical specialty (parsimonious model).

^d^ Multivariable model 4 adjusted for age group, type of medical institution, and department (parsimonious model).

Dermatology was associated with a significantly higher risk of discontinuation than that observed with internal medicine (multivariable 3: HR = 1.40, 95% CI 1.11–1.78, *p* = 0.005), a finding consistent across all models. However, given the substantial imbalance in sample sizes across biologic classes and specialties, and the presence of crossing Kaplan–Meier curves, these findings should be interpreted as descriptive associations rather than evidence of comparative superiority. Institution size also influenced retention outcomes, with higher discontinuation rates in smaller clinics and hospitals than in tertiary centers. These findings suggest specialty‐ and institution‐level variation in real‐world biologic prescribing and retention patterns. No comorbidities, including hypertension or diabetes, were significantly associated with the outcomes.

## 4 Discussion

In Korea, PsA treatment with biologic agents involves multiple specialists. In this study, 63.0% of PsA diagnoses and 80.2% of biologic prescriptions originated from dermatology departments, demonstrating that dermatology plays a central role in the diagnosis and treatment of PsA in Korea. IL‐17i and IL‐23i constituted the majority of first‐line biologic agents and were primarily prescribed by dermatologists, contrasting with internal medicine, where TNFi use was predominant. The risk of biologic discontinuation was significantly higher in dermatology than in internal medicine and higher in general hospitals, hospitals, and clinics than in tertiary hospitals. This study provides valuable insights into PsA management in Korea and underscores the need for standardized clinical guidelines across specialties to minimize specialty‐driven variability in PsA management.

A key finding of this study is the pronounced disparity between dermatology and internal medicine departments in both diagnosis and treatment of PsA. Whereas PsA diagnoses and biologic prescriptions within internal medicine remained relatively stable throughout the study period, those in dermatology rose steadily and disproportionately. Between 2011 and 2020, only 309 out of 788 patients diagnosed with PsA in internal medicine (39.2%) received biologic therapy, a rate consistent with prior studies, in which biologic use rarely exceeded 60% [11, 12, 13, 14]. Conversely, 1389 out of 1515 patients diagnosed in dermatology (91.7%) received biologics, indicating a substantially lower threshold for biologic initiation. This divergence indicates potential differences in diagnostic criteria, treatment decision‐making, and policy adherence in dermatology‐led PsA care [15, 16]. This divergence likely reflects differences in patient case‐mix, diagnostic emphasis, and specialty‐specific prescribing culture rather than simple non‐adherence to guidelines. These findings highlight the complexity of real‐world PsA management across specialties and underscore the value of cross‐specialty dialogue in developing consistent, context‐appropriate treatment approaches.

Although PsA remains a rare intractable disease in Korea, its prevalence has increased over the past decade [4, 17]. Given that more than a decade has passed since nationwide health insurance coverage expanded access to biologic therapy for PsA [18], the rapid increase in prevalence is unlikely to be explained solely by epidemiological factors. Until the underlying causes of this trend are clarified, caution is warranted when interpreting findings based only on nationwide claims data. In large‐scale cohort studies using health insurance databases, broad or nonspecific inclusion criteria are often applied to ensure adequate case numbers [19, 20]. However, if the clinical diagnosis of PsA is prone to variation or misclassification, for instance through inconsistent application of reimbursement indications, key epidemiological measures, including incidence, prevalence, and treatment rates, may be substantially biased. This limitation casts doubt on the reliability of national estimates regarding PsA burden, biologic prescription patterns, and downstream research on outcomes such as malignancy or comorbidities [21, 22, 23, 24].

Using cumulative national data, Bang et al. previously reported that only 11% of patients with PsA received biologics [4]. However, their analysis included patients diagnosed before biologic agents became widely accessible in Korea. Conversely, our study emphasized a more recently diagnosed cohort (2011–2022), a period characterized by substantial expansion in biologic use. Of the 2406 patients included, 1732 (71.9%) received biologic therapy, a proportion far exceeding previous reports. This pronounced increase indicates a substantial shift in treatment patterns, particularly after 2016, and may reflect both improved access and potential overutilization within current diagnostic and reimbursement systems. These patients likely represent a distinct treatment‐era subgroup with unique therapeutic characteristics compared with earlier cohorts, underscoring the need for stratified analyses. To improve the validity of claims‐based research, future studies must employ stricter inclusion criteria and foster cross‐specialty collaboration in validating diagnostic definitions and treatment patterns.

Internal medicine departments demonstrated a significantly lower hazard ratio for biologic discontinuation than did dermatology departments, though the underlying reasons cannot be determined from claims data alone [17]. Notably, dermatologists predominantly prescribed IL‐17 and IL‐23 inhibitors and often initiated biologic therapy on the same day as PsA diagnosis (median time to initiation: 0 days), whereas TNF inhibitors were initiated much later (median: 157 days). This pattern may partly reflect prior dermatologic management of psoriasis rather than de novo biologic initiation for PsA, as psoriasis typically precedes joint involvement by several years [25, 26, 27]. Nevertheless, these specialty‐related differences in treatment timing and drug selection highlight the potential value of multidisciplinary collaboration and consistent application of evidence‐based criteria across specialties [25, 26, 27].

Drug retention reflects not only treatment efficacy and safety but also patient‐related and external factors, including adherence, preferences, institutional formularies, and regional access [28, 29]. Accordingly, a higher retention rate does not necessarily imply a superior drug. Although IL‐23i and IL‐17i have demonstrated relatively longer retention times in several psoriasis‐focused studies [28, 29, 30], findings have been inconsistent, warranting cautious interpretation. In our study, IL‐23i also demonstrated the highest retention; however, this may partly reflect the disproportionately large number of prescriptions for this class, introducing potential bias. The primary aim of this analysis was not to determine the most effective biologic but to elucidate differences in prescribing patterns across specialties. From this perspective, retention patterns may reflect distinct specialty‐specific preferences.

Tertiary hospitals demonstrated significantly higher biologic retention rates than other healthcare institutions, likely attributable to the concentration of PsA specialists, multidisciplinary care coordination, and streamlined administrative support for biologic use. Such centers typically provide timely treatment adjustments and patient education, both of which enhance long‐term adherence. Conversely, smaller institutions may have limited clinical expertise or infrastructure capacity to support sustained disease management. These findings underscore the critical influence of institutional factors on biologic utilization and highlight the need to extend best practices across all tiers of care.

Some limitations should be considered. First, caution is warranted when interpreting drug retention rates, as both patient numbers and the preferred biologic classes vary considerably across medical specialties. Although TNFi were used with relative balance across departments, IL‐17i and IL‐23i prescriptions were predominantly concentrated in dermatology, with limited prescriptions in internal medicine. This imbalance complicates interdepartmental comparisons and warrants careful interpretation. Second, the analysis only included patients diagnosed with PsA after 2011, when biologics became more widely accessible in Korea. Consequently, patients diagnosed before this period were excluded, potentially limiting the generalizability of our findings. Third, the HIRA claims database does not capture biologic prescriptions issued outside the national insurance system (e.g., non‐reimbursed or self‐paid biologics), potentially resulting in underestimation of overall biologic use. Fourth, this study focused exclusively on the first biologic agent prescribed to each patient. Including second‐ or third‐line biologic treatments could introduce additional complexity to the classification and interpretation. Fifth, because of the structure of claims data, prescriptions issued by rheumatologists are frequently categorized as internal medicine. However, considering the use of specific diagnostic and rare disease codes, it is reasonable to infer that most of these prescriptions originated from rheumatology specialists. Sixth, the 180‐day gap criterion used to define discontinuation is consistent with prior Korean claims‐based studies on biologic retention in inflammatory arthritis [31]. However, this threshold may influence retention estimates; a shorter gap would capture more discontinuation events, whereas a longer gap may overestimate persistence. These considerations should be borne in mind when interpreting the retention analyses. Finally, patients coded with M07.1 (arthritis mutilans) were excluded because its extremely low prevalence precluded a statistically meaningful subgroup analysis. Moreover, its markedly different clinical course and prognosis compared with other PsA subtypes could increase heterogeneity within the study population and potentially bias the interpretation of results.

## 5 Conclusion

This nationwide analysis of the HIRA data revealed clear specialty‐specific patterns in the diagnosis and biologic treatment of PsA in Korea. Dermatology accounted for the majority of PsA diagnoses and biologic prescriptions, with a strong preference for IL‐17 and IL‐23 inhibitors, whereas internal medicine demonstrated more conservative biologic use and lower hazard ratios for discontinuation in adjusted models. The marked differences in treatment timing, drug selection, and retention patterns across specialties reflect the heterogeneity of real‐world PsA management and highlight the potential benefit of cross‐specialty collaboration and consistent application of evidence‐based approaches.

## Author Contributions

Conceptualization: Bon San Koo. Methodology: Bon San Koo, Ye‐Jee Kim. Data curation: Ye‐Jee Kim. Formal analysis: Bon San Koo, Ye‐Jee Kim, Yeo‐Jin Lee. Investigation: Bon San Koo, Ye‐Jee Kim, Yeo‐Jin Lee. Validation: Bon San Koo, Ye‐Jee Kim, Yeo‐Jin Lee. Visualization: Bon San Koo, Ye‐Jee Kim. Writing – original draft: Bon San Koo. Writing – review and editing: All authors. Supervision: Bon San Koo, Yong‐Gil Kim, Tae‐Hwan Kim. Project administration: Bon San Koo, Yong‐Gil Kim. Funding acquisition: Bon San Koo, Tae‐Hwan Kim.

## Funding

This work was supported by the National Research Foundation of Korea (NRF) grant funded by the Korean government (MSIT; No. NRF‐2021R1C1C1009815) and by a grant from the Patient‐Centered Clinical Research Coordinating Center (PACEN) funded by the Ministry of Health and Welfare, Republic of Korea (South Korea) (grant number: RS‐2021‐KH120130).

## Ethics Statement

This study was reviewed by the Institutional Review Board of Inje University Ilsan Paik Hospital (ISPAIK 2023‐11‐003‐001) according to the ethical principles of the Declaration of Helsinki. All patient information was anonymized, and the processed data were provided by HIRA. Consequently, informed consent was not required.

## Conflicts of Interest

The authors declare no conflicts of interest.

## Supporting information


**Figure S1:** Specialty‐based comparison of biologic drug classes using Kaplan–Meier curves (Overall, Internal Medicine, and Non‐Internal Medicine departments).
**Table S1:** Operational definitions of inclusion/exclusion criteria, outcome, and comorbidities (ICD‐10 codes and HIRA definitions).
**Table S2:** Medication codes for tumor necrosis factor $\alpha$ inhibitors, interleukin‐17 inhibitors, and interleukin‐23 inhibitors.
**Table S3:** Median survival time of the persistence of biologics stratified by medical specialty.

## Data Availability

The de‐identified health insurance claims data that support the findings of this study are available from the Health Insurance Review and Assessment Service (HIRA) under license and with regulatory approval. Researchers seeking access for public‐interest purposes must submit a formal application to HIRA via its designated data request form and pay the applicable processing fees. Data will be released only upon approval of the request. Please contact HIRA directly rather than the authors for all data access inquiries. Aggregate data outputs and the analytic code used in this study are available from the corresponding author upon reasonable request and are subject to HIRA's sharing policies.
