# Supplementary material for: Specialty‐Based Disparities in Biologic Use and Retention in Psoriatic Arthritis: A Nationwide Korean Claims Analysis
Source: Int J Rheum Dis. 2026 Jun 26;29(7):e70756. doi: 10.1111/1756-185x.70756 (PMC13309520; doi:10.1111/1756-185x.70756)

Supplementary materials

Table S1. Operational definitions of inclusion/exclusion criteria, outcome, and comorbidities

| Category | Disease | ICD-10 codes | Number of diagnosis/additional definition |
| --- | --- | --- | --- |
| Inclusion | Psoriatic arthritis (PsA) | M070, M072, M073 | V237 (rare incurable disease registration code) |
| Exclusion | Systemic lupus erythematosus | M32 |  |
|  | Rheumatoid arthritis | M05, M06 |  |
|  | Bechet’s disease | M352 |  |
|  | Ankylosing spondylitis | M45 |  |
|  | Inflammatory bowel disease | K51, K50 |  |
| Comorbidities | Charlson comorbidity index |  | ref. Quan 2005 |
|  | Diabetes Mellitus | E10-E14 | Admission or outpatient clinic ≥ 1 within a year |
|  | Dyslipidemia | E78 | Admission or outpatient clinic ≥ 1 within a year |
|  | Hypertension | I10-I13, I15 | Admission or outpatient clinic ≥ 1 within a year |
|  | Ischemic heart disease | I20-25 | Admission or outpatient clinic ≥ 1 within a year |
|  | Stroke | I63, I64, I693, I694, G459 | Admission or outpatient clinic ≥ 1 within a year |
|  | COPD | J44 | Admission or outpatient clinic ≥ 1 within a year |
|  | Asthma | J45-46 | Admission or outpatient clinic ≥ 1 within a year |

Table S2. Medication codes

| Category | Therapeutic class (Prescription duration) | General name | HIRA formulary code (9 digit) | Additional definition |
| --- | --- | --- | --- | --- |
| Medications | Tumor necrosis factor α inhibitors (TNFi) | Adalimumab | 488431BIJ, 488433BIJ, 488430BIJ, 488401BIJ, 488432BIJ |  |
|  |  | Etanercept | 455830BIJ, 455831BIJ, 455803BIJ, 455802BIJ, 455801BIJ |  |
|  |  | Golimumab | 621230BIJ, 621231BIJ, 621232BIJ |  |
|  |  | Infliximab | 383502BIJ, 383501BIJ, 687001BIJ |  |
|  | Interleukin-17 inhibitor (IL-17i) | Secukinumab | 644602BIJ, 644603BIJ |  |
|  |  | Ixekizumab | 667801BIJ |  |
|  | Interleukin-12/23 inhibitor (IL-23i) | ustekinumab | 615032BIJ, 615030BIJ, 615031BIJ |  |
|  | Interleukin-23 inhibitor | guselkumab | 670901BIJ |  |
|  |  | risankizumab | 686202BIJ, 686201BIJ |  |

Table S3. Median survival time of the persistence of biologics = Time of 50% discontinuation (day)

|  | Total | TNFi | IL-17i | IL-23i |
| --- | --- | --- | --- | --- |
| Total, number of patients | 1732 | 279 | 380 | 1073 |
| median [lower, upper] | 709 [680, 750] | 460 [381, 607] | 476.5 [435, 572] | 624 [589, 675] |
| Internal medicine, number of patients | 192 | 168 | 20 | 4 |
| median [lower, upper] | 456 [385, 693] | 601.5 [430, 795] | 348 [253, 456] | 1 [1, NA] |
| Demartology, number of patients | 1509 | 111 | 359 | 1039 |
| median [lower, upper] | 596 [554, 631] | 363 [263, 518] | 547 [452, 598] | 639 [603, 692] |
| Others, number of patients | 31 | 0 | 1 | 30 |
| median [lower, upper] | 197 [99, 566] |  | 388 [NA, NA] | 169 [99, 566] |
| Non-internal medicine, number of patients | 1540 | 111 | 360 | 1069 |
| median [lower, upper] | 588.5 [550, 624] | 363 [263, 518] | 543 [452, 591] | 626 [589, 680] |

Figure S1. Specialty-based comparison of biologic drug classes. Kaplan–Meier curves illustrating drug survival of different biologic classes stratified by specialty. (A) Overall comparison of biologic classes across all specialties. (B) Within internal medicine departments. (C) Within non-internal medicine departments.


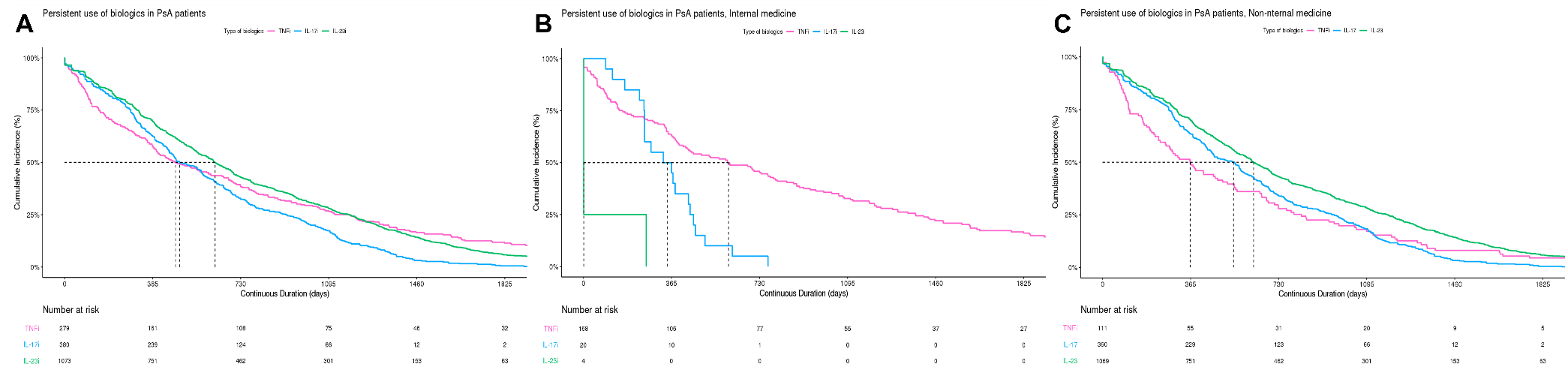

Supplement: Supplementary file 1 — Figure S1: Specialty‐based comparison of biologic drug classes using Kaplan–Meier curves (Overall, Internal Medicine, and Non‐Internal Medicine departments). Table S1: Operational definitions of inclusion/exclusion criteria, outcome, and comorbidities (ICD‐10 codes and HIRA definitions). Table S2: Medication codes for tumor necrosis factor $\alpha$ inhibitors, interleukin‐17 inhibitors, and interleukin‐23 inhibitors. Table S3: Median survival time of the persistence of biologics stratified by medical specialty. [file APL-29-e70756-s001.docx]
